# Supplementary material for: Nutritional Assessment of Lactarius drassinus and L. controversus from the Cold Desert Region of the Northwest Himalayas for Their Potential as Food Supplements
Source: J Fungi (Basel). 2023 Jul 20;9(7):763. doi: 10.3390/jof9070763 (PMC10381459; doi:10.3390/jof9070763)
Supplement: Supplementary file 1 [file jof-09-00763-s001.zip › Supplementary Material S1.pdf]

Supplementary Material S1

Nutritional Assessment of *Lactarius drassinus* and *L. controversus* from Cold Desert Region of Northwest Himalayas for Their Potential as Food Supplements

Hom-Singli Mayirnao[a], E-mail address: homsingli08@gmail.com
Samta Gupta[a] E-mail address: guptasamta0203@gmail.com
Sarda Devi Thokchom[a], E-mail address: srd17thkchm@gmail.com
Karuna Sharma[a], E-mail address: sharmak210596@gmail.com
Tahir Mehmood[b], E-mail address: mehmoodt898@gmail.com
Surinder Kaur[c], E-mail address: surinder@sgtbkhalsa.du.ac.in
Yash Pal Sharma[b], E-mail address: yashdbm3@yahoo.co.in
Rupam Kapoor[a*] E-mail address: kapoor_rupam@yahoo.com

[a]Department of Botany, University of Delhi, Delhi-110007, India
[b]Department of Botany, University of Jammu, Jammu- 180016, India.
[c]SGTB Khalsa College, University of Delhi, Delhi-110007, India

[*]Corresponding author

1. **Collection of mushrooms**

Systematic collection of mushrooms was undertaken in August, 2021. Two wild edible mushrooms, species 1 and species 2, were collected from Tesbo (34°25′47.0″ N, 75°49′13.5″ E) and Holiyal (34°25′57.5″ N, 75°45′58.5″ E) respectively. Field equipment such as digital camera, scale, trowel, heater, and heat convector card board, were used; and chemical reagents namely, 30% potassium hydroxide, 10% ferrous sulphate, 2% phenol, and guaiacol were used for biochemical tests. Methuen Handbook of Colour was followed for colour indications. Collection of samples was made in the morning and field characteristics of mushrooms were recorded in the data sheet (mushroom description form). Simultaneously, biochemical spot tests were performed on the surface and context of pileus and stipe in freshly collected samples and other necessary processing such as heat-drying and packaging for preservation were carried out.

Macromorphological characterization of *L. drassinus*

Description

Pileus 74–125 mm diam., convex to plano-convex with broadly depressed centre, gradually becoming infundibuliform; surface smooth, dry, shiny, dull white to light grey (3C2), brownish grey (4D2), turning cream white (7A2), faintly zonate; sometimes with white tomentum when young, never peeling easily; sometimes cracked towards the margin, brittle in consistency; margin inrolled to incurved. Gills 2–4 mm broad, decurrent to subdecurrent, rather crowded (11–15/cm including lamellulae), rarely anastomosing, reddish grey (7B2), pinkish white (8A2), edges entire; lamellulae present in 3–4 lengths. Stipe 15–38 × 22–30 mm, central, cylindrical, sometimes slightly swollen at the base, dry, white to dull white. Context moderately thick at pileus, white, solid in stipe, turning yellowish white (1A2), 30% KOH, deep red (10C8) with guaiacol, olive (1E3–4) with $FeSO_4$. Latex copious, white, greyish yellow (2C4–5) with KOH. Taste mild. Odour pleasant.

Distribution and habitat

Solitary to scattered, mixed growth stage, growing in associations with *Salix alba* and *Populus* sp.

Macromorphological characterization of *L. controversus*

Description

Pileus 53–120 mm diam., when young, plano-convex with slightly depressed centre when mature, surface smooth, dry, slightly rugulose, reddish blonde (5C4) when young, dull white to light grey (3C2), brownish grey (4D2), margin entire to undulate. Lamellae subdecurrent to broadly adnate, crowded (20–22/cm including lamellulae), 4–5 mm wide, yellowish white to light yellow (4A2–4) when young, light orange (5A5) to rust-orange (5C5) when mature, orange to deep orange (6A7–8) when bruised, margins offwhite (4A2); lamellulae numerous, unevenly distributed, 3–4 tiers. Stipe 40–65 × 6–13 mm, cylindrical, equal, sometimes tapering toward base, dry, central,

brittle, concolorous with the pileus, sometimes white at base. Context yellowish white (4A2), pale to light orange (6A4–5) on exposure; stuffed in stipe, brownish red (8D7–8) in guaiacol, light yellowish in KOH and unchanging in $FeSO_4$; taste mild, odour mild. Latex copious, white, turning pink (8A4) on exposure. Taste mild. Odour pleasant.

Distribution and habitat

Solitary to scattered, mixed growth stage, growing in associations with *Salix alba*.
