# Supplementary material for: Nutritional Assessment of Lactarius drassinus and L. controversus from the Cold Desert Region of the Northwest Himalayas for Their Potential as Food Supplements
Source: J Fungi (Basel). 2023 Jul 20;9(7):763. doi: 10.3390/jof9070763 (PMC10381459; doi:10.3390/jof9070763)
Supplement: Supplementary file 1 [file jof-09-00763-s001.zip › Supplementary Material S2.pdf]

Supplementary Material S2

Nutritional Assessment of *Lactarius drassinus* and *L. controversus* from Cold Desert Region of Northwest Himalayas for Their Potential as Food Supplements

Hom-Singli Mayirnao[a], E-mail address: homsingli08@gmail.com

Samta Gupta[a] E-mail address: guptasamta0203@gmail.com

Sarda Devi Thokchom[a], E-mail address: srd17thkchm@gmail.com

Karuna Sharma[a], E-mail address: sharmak210596@gmail.com

Tahir Mehmood[b], E-mail address: mehmoodt898@gmail.com

Surinder Kaur[c], E-mail address: surinder@sgtbkhalsa.du.ac.in

Yash Pal Sharma[b], E-mail address: yashdbm3@yahoo.co.in

Rupam Kapoor[a*] E-mail address: kapoor_rupam@yahoo.com

[a]Department of Botany, University of Delhi, Delhi-110007, India

[b]Department of Botany, University of Jammu, Jammu- 180016, India.

[c]SGTB Khalsa College, University of Delhi, Delhi-110007, India

[*]Corresponding author

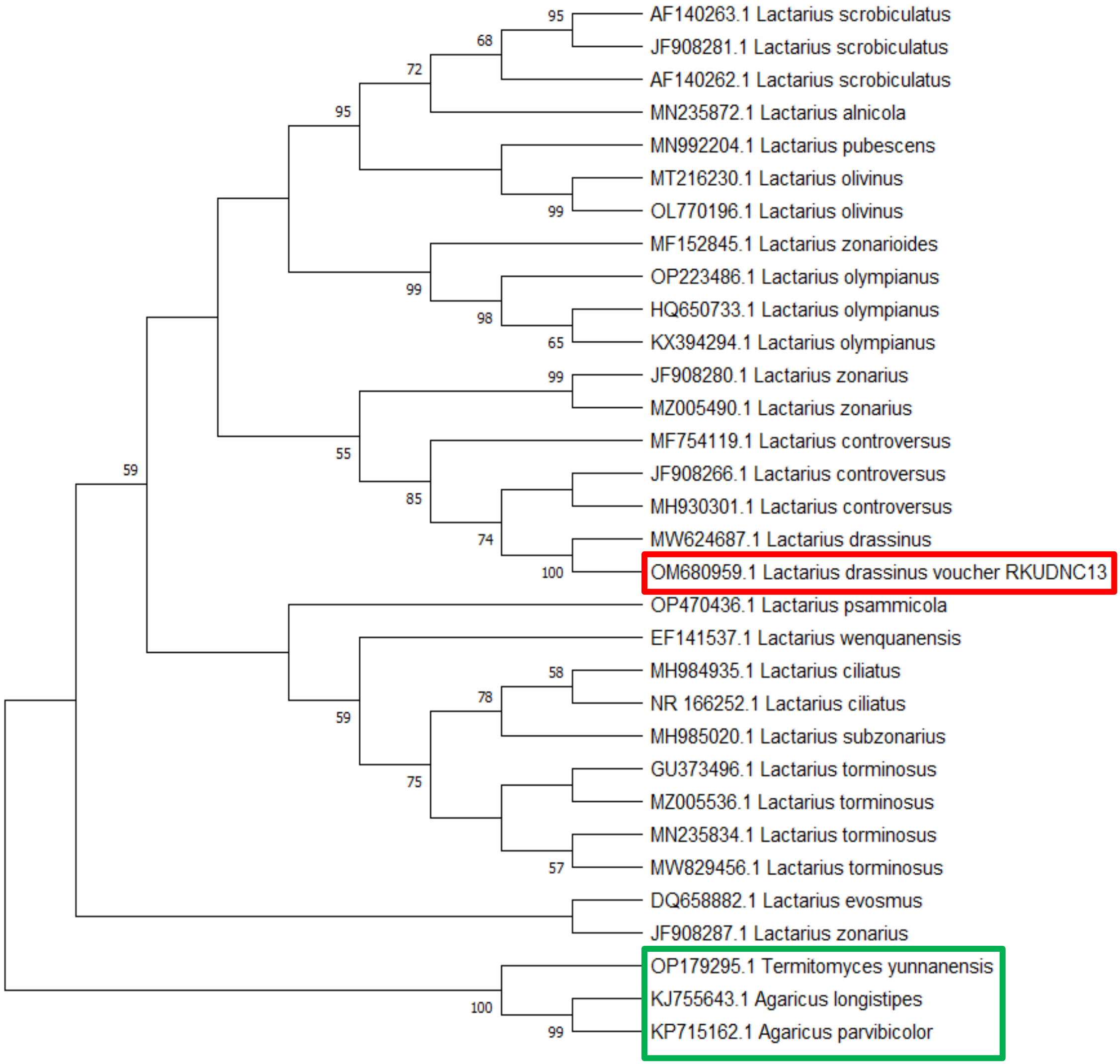

Figure S1. Neighbor-Joining tree constructed using sequences of internal transcribed spacer (ITS) region of the rRNA of *Lactarius* spp. The bootstrap consensus tree inferred from 1000 replicates is taken to represent the evolutionary history of the taxa analyzed. Branches corresponding to partitions reproduced in less than 50%bootstrap replicates are collapsed. The evolutionary distances were computed using the Kimura 2-parameter method and are in the units of the number of base substitutions per site. The rate variation among sites was modeled with a gamma distribution. *L. drassinus* is highlighted in red and the outgroup (*Termitomyces yunnanensis*, *Agaricus longistipes*, and *A. parvibicolor*) in green.

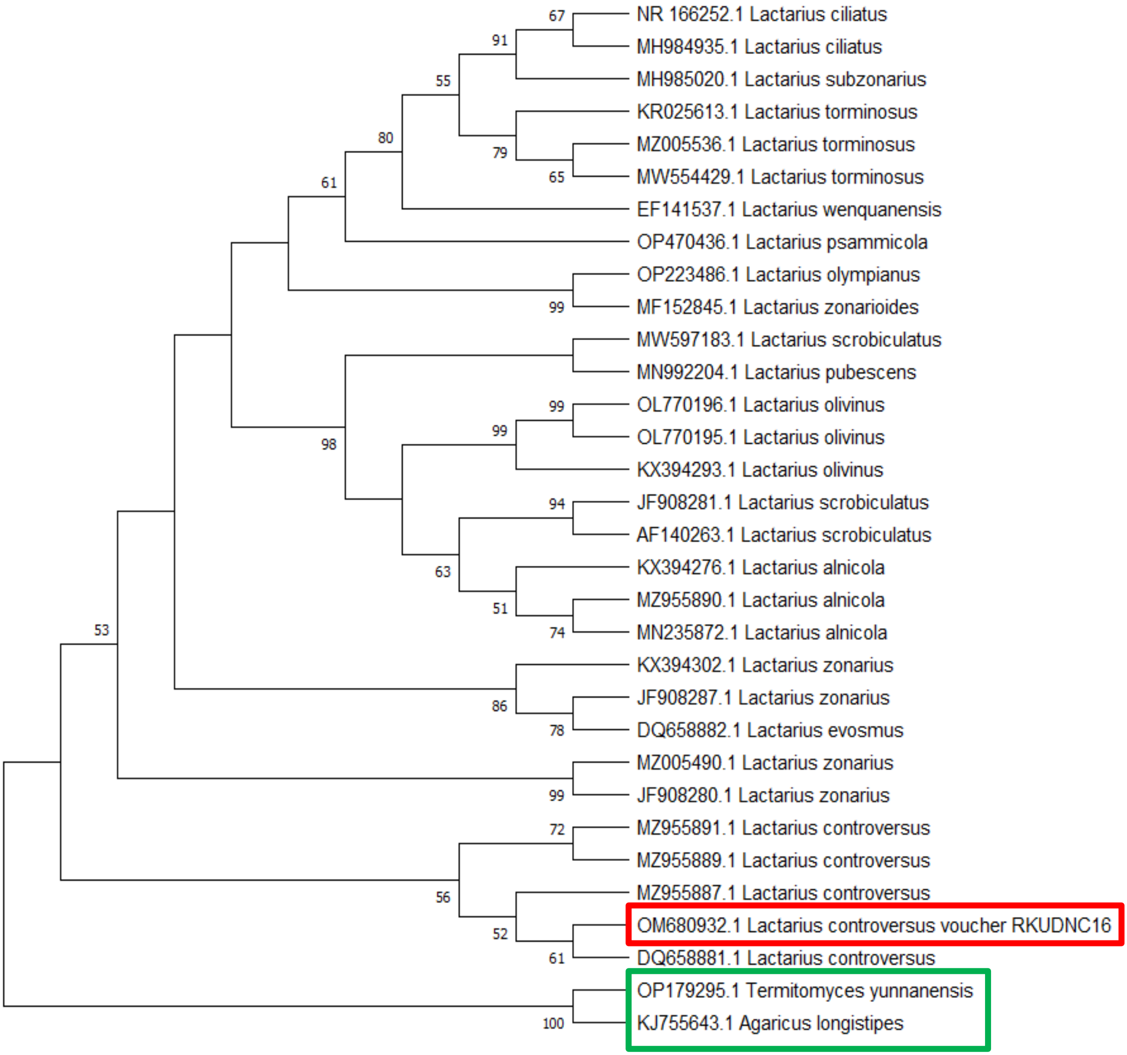

Figure S2. Neighbor-Joining tree constructed using sequences of internal transcribed spacer (ITS) region of the rRNA of *Lactarius* spp. The bootstrap consensus tree inferred from 1000 replicates is taken to represent the evolutionary history of the taxa analyzed. Branches corresponding to partitions reproduced in less than 50%bootstrap replicates are collapsed. The evolutionary distances were computed using the Kimura 2-parameter method and are in the units of the number of base substitutions per site. The rate variation among sites was modeled with a gamma distribution. *L. controversus* is highlighted in red and the outgroup (*Termitomyces yunnanensis* and *Agaricus longistipes*) in green.
